# Votucalis, a Novel Centrally Sparing Histamine-Binding Protein, Attenuates Histaminergic Itch and Neuropathic Pain in Mice

**DOI:** 10.3389/fphar.2022.846683

**Published:** 2022-03-08

**Authors:** Ibrahim Alrashdi, Amal Alsubaiyel, Michele Chan, Emma E. Battell, Abdel Ennaceur, Miles A. Nunn, Wayne Weston-Davies, Paul L. Chazot, Ilona Obara

**Affiliations:** ^1^ School of Pharmacy, Newcastle University, Newcastle-upon-Tyne, United Kingdom; ^2^ Translational and Clinical Research Institute, Newcastle University, Newcastle-upon-Tyne, United Kingdom; ^3^ School of Pharmacy, University of Sunderland, Sunderland, United Kingdom; ^4^ Akari Therapeutics Plc, London, United Kingdom; ^5^ Department of Biosciences, Durham University, Durham, United Kingdom

**Keywords:** Votucalis, histamine-binding protein, histamine receptor, itch, neuropathic pain, CNS-sparing, mice

## Abstract

Votucalis is a biologically active protein in tick (*R. appendiculatus*) saliva, which specifically binds histamine with high affinity and, therefore, has the potential to inhibit the host’s immunological responses at the feeding site. We hypothesized that scavenging of peripherally released endogenous histamine by Votucalis results in both anti-itch and anti-nociceptive effects. To test this hypothesis, adult male mice were subjected to histaminergic itch, as well as peripheral nerve injury that resulted in neuropathic pain. Thus, we selected models where peripherally released histamine was shown to be a key regulator. In these models, the animals received systemic (intraperitoneal, i.p.) or peripheral transdermal (subcutaneous, s.c. or intraplantar, i.pl.) administrations of Votucalis and itch behavior, as well as mechanical and thermal hypersensitivity, were evaluated. Selective histamine receptor antagonists were used to determine the involvement of histamine receptors in the effects produced by Votucalis. We also used the spontaneous object recognition test to confirm the centrally sparing properties of Votucalis. Our main finding shows that in histamine-dependent itch and neuropathic pain models peripheral (s.c. or i.pl.) administration of Votucalis displayed a longer duration of action for a lower dose range, when compared with Votucalis systemic (i.p.) effects. Stronger anti-itch effect was observed after co-administration of Votucalis (s.c.) and antagonists that inhibited peripheral histamine H_1_ and H_2_ receptors as well as central histamine H_4_ receptors indicating the importance of these histamine receptors in itch. In neuropathic mice, Votucalis produced a potent and complete anti-nociceptive effect on mechanical hypersensitivity, while thermal (heat) hypersensitivity was largely unaffected. Overall, our findings further emphasize the key role for histamine in the regulation of histaminergic itch and chronic neuropathic pain. Given the effectiveness of Votucalis after peripheral transdermal administration, with a lack of central effects, we provide here the first evidence that scavenging of peripherally released histamine by Votucalis may represent a novel therapeutically effective and safe long-term strategy for the management of these refractory health conditions.

## Introduction

Histamine [2-(4-imidazolyl)-ethylamine], that can be found in almost all tissues of the mammalian body (Haas et al., 2008), is synthesised and stored primarily in cytosolic granules of the peripheral and central tissues, mast cells, basophils, eosinophils, platelets, basophiles, histaminergic neurons and enterochromaffin cells (Benly, 2015; Branco et al., 2018). Also, other types of non-professional histamine cells, such as epithelial cells, dendritic cells, neutrophils and T lymphocytes can synthesize and secrete histamine immediately after its production, although they do not store it (O’Mahony et al., 2011). Given its wide distribution in multiple cell types, histamine has been shown to regulate many physiologic and pathologic conditions, including pruritus/itch (Baron et al., 2001) and chronic pain (Obara et al., 2020). Histamine produces these regulatory effects *via* four G protein-coupled histamine receptors: H_1_, H_2_, H_3_ and H_4_ receptors that are expressed in both the central and peripheral nervous system (CNS and PNS, respectively) (Lindskog, 2017). Apart from distribution, histamine receptors differ in their pharmacological and signal transduction properties, and for that reason histamine has been shown to have different effects depending on the histamine receptor subtype it is bound to (Ikoma et al., 2006; Panula et al., 2015; Lindskog, 2017; Obara et al., 2020). For example, histamine depending on its concentration, site of action and type of receptors implicated, can both reduce and aggravate sensory perception of itch and pain (Tamaddonfard and Rahimi, 2004; Ikoma et al., 2006). Therefore, histamine as well as histamine receptors have long been attractive targets for therapeutic interventions in conditions where itch and pain are symptoms requiring treatment (O'Donoghue and Tharp, 2005; Obara et al., 2020). However, despite these multiple efforts, the understanding of itch signalling *via* histamine system is far from completely understood (Rossbach et al., 2011), and standard antihistamine treatments targeting H_1_ receptors showed limited efficacy (Patel and Yosipovitch, 2010). Also, findings reporting roles played by histamine in chronic pain are somewhat contradictory (Haas et al., 2008; Obara et al., 2020). Thus, the therapeutic potential of histamine modulation still requires clarification.

The discovery of histamine-binding proteins in the saliva of arthropods/ticks has provided a new and powerful tool to revisit the role of histamine in the regulation of itch and pain (Weston-Davies et al., 2005; Chmelař et al., 2019). Ticks are species of blood-feeding arthropods with animal and human hosts and have adopted a specialized strategy to suppress host-defense immunological mechanisms at the feeding site (Paesen et al., 1999; Ryffel et al., 2005). The saliva of the tick contains complex bioactive molecules, including a selection of proteins and lipids with anti-inflammatory, anti-coagulant, anti-platelet, anti-fibrotic, anti-hemostatic and immunomodulatory effects (Štibrániová et al., 2019). Despite the identification of a large number of highly bioactive tick salivary molecules, their development for medical purposes remains in its infancy. Studies have been performed on only a small number of tick salivary compounds, mainly proteins (Aounallah et al., 2020). Votucalis (also known as EV131 or HPB1), one of the proteins extracted from female *Rhipicephalus Appendiculatus* tick displays histamine binding properties and is linked to a broad ligand-binding protein family, known as lipocalins. Votucalis is a histacalin, which captures histamine within two different internal binding sites with the high-affinity binding site displaying a 100-fold higher affinity than H_1_ and H_2_ receptors, and a similar affinity to the H_3_ and H_4_ receptors (Paesen et al., 1999; Paesen et al., 2000). Interestingly, there are both pre-clinical and clinical studies showing therapeutic effects achieved by scavenging of endogenous histamine by recombinant Votucalis. These studies focused on conditions where histamine is recognized as an important inflammatory mediator and included models of acute respiratory distress syndrome (ARDS), asthma and allergic rhinitis (Ryffel et al., 2005).

This study further explored novel refractory therapeutic indications based on scavenging of peripherally released endogenous histamine by Votucalis and focused on mouse models of acute itch and chronic neuropathic pain. Histamine is a known key regulator of both conditions and we, therefore, hypothesized that selective targeting of peripheral histamine by Votucalis would result in both anti-pruritic and anti-nociceptive effects that may suggest novel and safe therapeutic approaches in the regulation and control of these conditions.

## Materials and Methods

### Subjects

*C57BL6/J mice.* Experimental protocols (PPL: P8E3496FA, P6694C943) were performed under UK Home Office license, with the Animal Welfare Ethical Review Body (AWERB) local approval, and in accordance with current UK legislation as defined in the Animals (Scientific Procedures) Act 1986. The Animal Research: Reporting of *In Vivo* Experiments (ARRIVE) has been followed in reporting this study.

Adult male C57BL/6J mice (8 weeks of age; 20–25 g; Charles River Laboratories, Kent, UK) were allowed to acclimatize to the colony room (Life Sciences Support Unit Durham University, UK and Comparative Biology Centre, Newcastle University, UK) for at least 7 days after arrival, and were housed in polyethylene cages (4 per cage), controlled for temperature (21°C) and humidity (55%) under a regular 12-h day/night cycle (lights on at 8:00 a.m.; lights off at 8:00 p.m.). Standard laboratory rodent chow and water were available *ad libitum*. Animals were habituated to testing procedures for at least 3–4 days before experiments. The handling and testing of the animals were conducted during the light phase, between 9:00 a.m. and 4:00 p.m. All efforts were made to minimize animal suffering and to reduce the number of animals used in the study.

### Material: Preparation and Administration

Votucalis was provided by Akari Therapeutics Plc (UK) as a stock solution of 5.8 mg ml^−1^ in phosphate-buffered saline and was stored at −80°C (histamine binding (K_D_) to Votucalis = 1.6 nM, data not shown). For all administrations, Votucalis was thawed immediately before injections and prepared in a vehicle (sterile saline; 0.9% NaCl; Fresenius Kabi Ltd., UK) solution at required concentrations, as described below. The isolation, cloning and detailed crystal structure of Votucalis were reported previously (Paesen et al., 1999; Paesen et al., 2000).

*Systemic i.p. administration.* Mice were weighed and randomised to receive either Votucalis or vehicle; they were injected i.p. with Votucalis at 1, 3, 10, 20 and 40 mg kg^−1^ body weight or vehicle (saline) solution as a control group. In the itch model, Votucalis/vehicle was injected 30 min before injection of pruritogens. In the neuropathic pain model, Votucalis/vehicle was administered once every 24 h on days 7, 8, 9 and 10 post induction of neuropathic pain.

*Peripheral s.c. administration.* Mice were weighed and randomised to receive either Votucalis or vehicle; they were injected s.c. with Votucalis at 0.3, 1, 3, 10 and 20 mg kg^−1^ body weight or vehicle (saline) solution as a control group. In the itch model, Votucalis/vehicle was injected once 30 min before injection of pruritogens. In the spontaneous object recognition task performed in naïve animals, Votucalis (20 mg kg^−1^ body weight) was injected once 30 min before the task, and then the injection was repeated every 24 h for a total of 4 days.

*Peripheral intraplantar (i.pl.) administration.* Mice were randomized to receive either Votucalis or vehicle; they were injected i.pl. with Votucalis (0.0075, 0.025, 0.075 and 0.25 mg paw^−1^) or equivalent vehicle (saline) solution without Votucalis as a control group. Injections were given over 1 min in a volume of 50 µL without anesthesia into the plantar surface of the animal hind paw ipsilateral to sciatic nerve injury. In the neuropathic pain model, Votucalis/vehicle was administered once every 24 h on days 7, 8, 9 and 10 post induction of neuropathic pain.

To determine the involvement of histamine receptors in the effects produced by Votucalis as well as to potentiate the anti-pruritic and anti-nociceptive effects produced by Votucalis, selective H_1_ receptor antagonist (mepyramine maleate, 10 and 20 mg kg^−1^; Sigma-Aldrich, UK), selective H_2_ receptor antagonist (ranitidine hydrochloride, 15 mg kg^−1^; Tocris Bioscience, UK) and selective H_4_ receptor antagonist (JNJ 7777120, 20 mg kg^−1^; Tocris Bioscience, UK) were co-injected with Votucalis or alone 30 min before injection of pruritogen. The antagonists were dissolved immediately before injections (i.p. or s.c.) in sterile saline (0.9% NaCl; Fresenius Kabi Ltd., UK), except JNJ 7777120 which was dissolved in DMSO (dimethyl sulfoxide, 5%; Sigma-Aldrich, UK). Control animals received equivalent vehicle (saline or 5% DMSO) injections. The concentration and timing of antagonist injections and measurements were based on previously published reports (Bell et al., 2004; Dunford et al., 2007; Rossbach et al., 2011).

### Itch Model

*Induction of itch.* As previously published (Sun et al., 2009; Obara et al., 2015), histamine-dependent itch was induced in mice by injection of compound 48/80 (100 μg; Sigma-Aldrich, UK), while histamine-independent itch was induced by injection of chloroquine (chloroquine diphosphate salt, 200 μg; Sigma-Aldrich, UK). Itch-inducing agents were dissolved in sterile saline and administered s.c. into the nape of the mouse neck in a volume of 50 μL. Prior to itch experiments, the back of the mouse neck was shaved, and animals were given 30 min to acclimatize to a small plastic chamber. For administration of pruritogens, mice were removed from the chamber, gently restrained, and injected. Itch behavior in the mouse that developed after s.c. injection of pruritogens was recorded with a digital video camera and analyzed.

*Behavioral assessment.* In all experiments, the observer was not aware of the doses and/or treatment administered. One scratch was defined as a lifting of the hind limb toward the injection site (the shaved area of the neck) and then replacing the limb back to the floor, regardless of how many scratching strokes (bouts of scratching) took place between those two movements (Shimada et al., 2006; Obara et al., 2011). The scratching was quantified as the total number of scratches across a 40-min observation period, or the cumulative number of scratches observed for 40 min at 5-min intervals. Each mouse was used only once, in one experiment.

### Neuropathic Pain Model

*Induction of neuropathic pain.* As previously published (Bennett and Xie, 1988; Obara et al., 2013), mice were subjected to peripheral neuropathy induced by chronic constriction injury (CCI) of the sciatic nerve. The injury was performed under isoflurane anesthesia delivered *via* a nose cone (up to 5% isoflurane with oxygen as the carrier gas for induction and 1.5–2.5% for maintenance). The skin on the lateral surface of the left thigh was shaved and an incision was made just below the left hipbone, parallel to the sciatic nerve. The *biceps femoris* and the *gluteus superficialis* were separated and the left sciatic nerve was exposed. Proximal to the sciatic trifurcation, the injury was produced by three loose ligations (4/0 silk) around the sciatic nerve. The ligatures were tied loosely around the nerve with 1 mm spacing, until they elicit a brief twitch in the respective hindlimb, which prevented the application of an excessively strong ligation. The total length of nerve affected was 3–4 mm. After the ligation, the muscle and skin were closed in two separate layers. In sham control mice, the sciatic nerve was exposed as described above but no contact was made with the nerve.

*Behavioral assessment.* In all experiments, the observer was not aware of the dose of Votucalis administered.

Mechanical hypersensitivity was assessed by measuring the withdrawal threshold of the mouse paw in response to mechanical stimuli using von Frey filaments in the 0.04–6.0 g range (Stoelting, Wood Dale, IL, USA). Mice were placed individually in a plastic cage (10 × 6 × 6 cm) with a metal mesh floor and were allowed to habituate before testing began. Animals were also habituated over a period of 3–4 consecutive days by recording a series of baseline measurements. The filaments were applied in ascending order of strength, each five times at an interval of 2–3 s to the plantar surface of the hind paw as described previously (Bourquin et al., 2006; Obara et al., 2011). The smallest filament eliciting a foot withdrawal response was considered the threshold stimulus. Data were collected from both the ipsilateral and contralateral paw to the side of the injury.

Thermal (heat) hypersensitivity (Hargreaves test) was assessed by measuring the latency of paw withdrawal response to a noxious thermal stimulus using a radiant heat-emitting device (IITC Life Science Inc., USA), as described previously (Hargreaves et al., 1988; Obara et al., 2011). Mice were placed individually in a plastic cage (12 × 10 × 10 cm) on an elevated glass platform and allowed to habituate to the apparatus before testing began. Animals were also habituated over a period of 3–4 consecutive days by recording a series of baseline measurements. A radiant heat source of constant intensity was applied to the plantar surface of the paw through the glass plate and the latency to paw withdrawal was measured. The hind paw received three stimuli and the inter-stimulus interval was at least 3–5 min to prevent injury. Withdrawal latencies were defined as the mean of the three readings for each hind paw. A cut-off of 20 s was employed to avoid tissue injury. Data was collected from both the ipsilateral and contralateral paw to the side of the injury.

Both mechanical hypersensitivity at the lateral plantar surface of the hind paw and heat hypersensitivity were assessed before nerve injury (as basal pain threshold) and then testing commenced on day 7 after the sciatic nerve injury and continued for three consecutive days with behavioral testing on day 7, 8, 9 and 10 post injury. Both tests were performed prior to administration of Votucalis (i.p. or i.pl.) and were repeated at 30 min, 1, 2, 4, 6, 8 and 24 h after each Votucalis administration on the four test days. Each animal first underwent von Frey testing followed by the Hargreaves test.

### Spontaneous Object Recognition Task

Short-term recognition memory was assessed using the spontaneous object recognition (SOR) task (Ennaceur and Delacour, 1987).

*Behavioral assessment.* In all experiments, the observer was not aware of the doses and/or treatment administered. The animals received a total of four 12-min habituation sessions. During the first 3 days of habituation, mice were placed individually in a matte black wooden box (60 × 60 × 60 cm) and allowed to explore the apparatus for 12 min. On the 4th day of habituation, mice were exposed to an object placed in the middle of the apparatus. The animals did not re-encounter this object afterwards, during the object recognition test.

The task consisted of an exposure and test phase, each lasting 3 min. During the exposure phase, mice were exposed to an identical pair of objects (A_1_ and A_2_), placed at the back-left and back-right corner of the wall, about 4 cm from the wall. Following a 1-h delay, mice were placed back into the apparatus to explore a copy of the familiar object (A_3_) and a novel object (B). Each object was available in triplicates and the objects were cleaned with 3% hydrogen peroxide solution (EndoSan, UK) between animals to avoid unwanted olfactory cues. The location and order in which the novel objects presented were counterbalanced. Exploratory behavior was recorded by an overhead video camera on one side of the apparatus. The animals were tested for recognition memory on the 1st and 4th day of treatment with Votucalis (s.c.). The effects of Votucalis were compared to mepyramine maleate (10 mg kg^−1^ body weight, i.p.) and vehicle (saline, i.p.).

Object exploration was defined as the nose of the mouse being directed towards the object at less than 1 cm. Novelty preference was measured by dividing the difference score (difference score: novel minus familiar) with the total exploration time. This results in the discrimination ratio ranging between -1 and 1, with a negative score indicating a preference to the familiar object and a positive score indicating a preference for the novel object (Ennaceur and Delacour, 1988).

### Data and Statistical Analysis

All data were recorded with Microsoft Excel. Itch data were collected as videos. Mice were randomly assigned to experimental groups. Each group included 5–18 mice, however the exact group size for each experimental group/condition is indicated in the figure legend for each figure. Data analysis and statistical comparisons were performed using GraphPad PrismTM, version 7.00 for Windows/OS (GraphPad Software, CA, USA, www.graphpad.com). Statistical analysis was performed by one- or two-way ANOVA with Bonferroni’s multiple comparison post-hoc tests or by unpaired Student’s t-test when two groups were compared. A value of *p* < 0.05 was considered to be statistically significant. Results are presented as mean ± standard error of the mean (SEM).

## Results

### Systemic Administration of Votucalis Reduced Histaminergic, but Not Non-histaminergic, Itch Behavior

**FIGURE 1:**
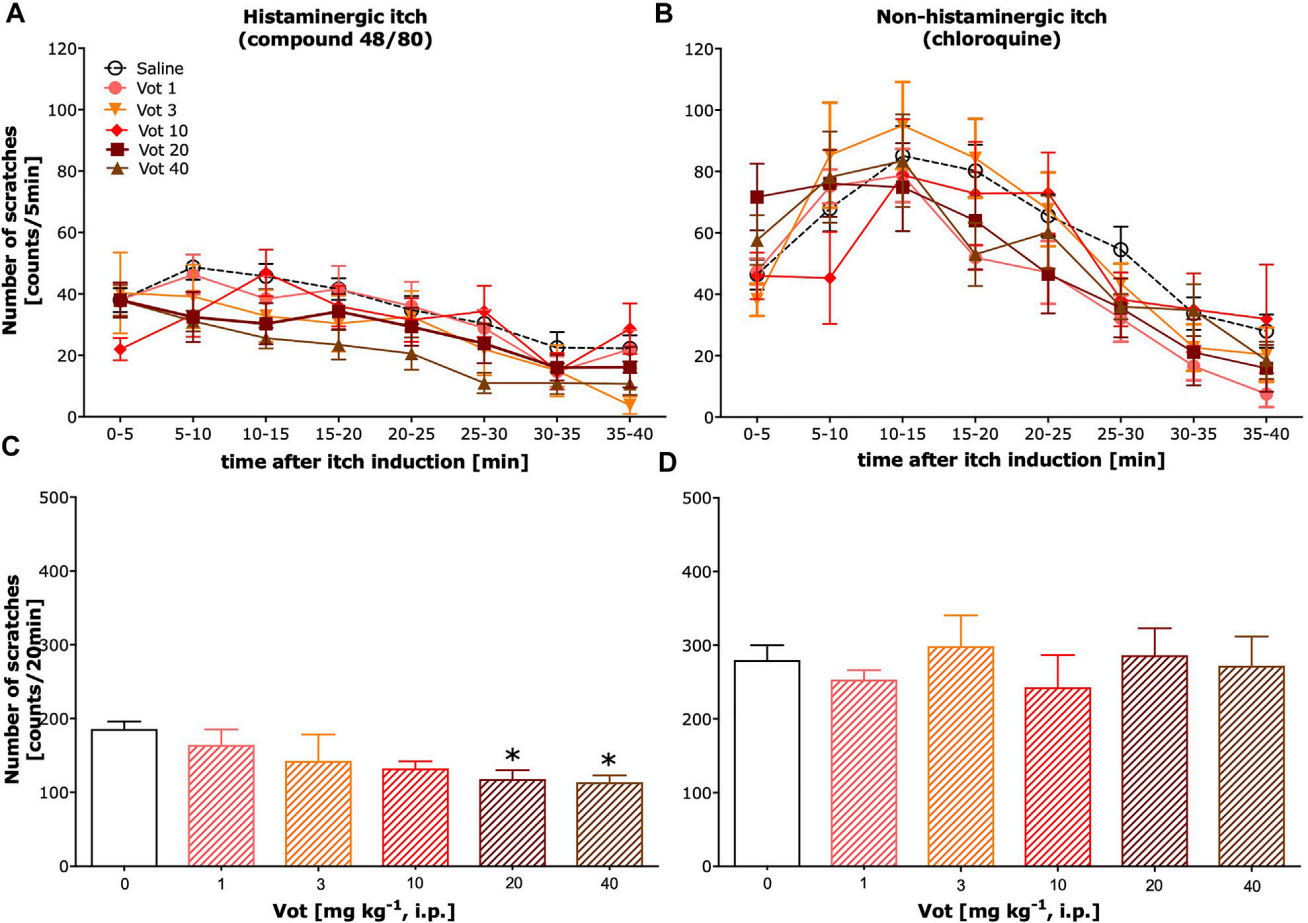
Systemic administration of Votucalis inhibited itch behaviour induced by histamine-dependent, but not histamine-independent pruritogens, in mice. **(A,B)** Time-course effect of systemic (i.p.) injection of Votucalis (Vot), or vehicle (saline), on the total number of scratches that occurred over a 40-min time period after injection of compound 48/80 **(A)** or chloroquine **(B)** into the nape of the mouse neck. Mice received an injection of Votucalis (1–40 mg kg^−1^; *n* = 6–9) or vehicle (saline; *n* = 14–18) 30 min before the injection of pruritic agents. Itch behaviour was recorded, and scratches were counted in 5-min intervals for 40 min **(C,D)** Bar graphs displaying the total number of scratches across the first 20-min observation period after injection of compound 48/80 **(C)** or chloroquine **(D)** for each treatment. Data are presented as means ± SEM; the asterisk (*) denotes significance vs. vehicle control animals; *p < 0.05 (one-way ANOVA, followed by Bonferroni’s comparison post-hoc test).

Administration (s.c.) of both compound 48/80 and chloroquine induced scratching behaviour that lasted for ∼40 min (Figures 1A,B respectively; total number of scratches in the vehicle control group for compound 48/80: 283.9 ± 23.4 and for chloroquine: 461.2 ± 30.2). Dose-dependent significant inhibition of itch behaviour resulting from systemic (i.p.) treatment with Votucalis was only observed in histamine-dependent itch induced by compound 48/80 (Figure 1A; drug effect: F_(5,339)_ = 6.4, *p* < 0.0001). The anti-itch effect produced by Votucalis was not observed for the whole 40 min of the observation period, when compared with vehicle control animals (*p* > 0.05). As indicated in Figure 1C, systemic Votucalis promoted significant inhibition of histamine-dependent itch within the first 20 min from the induction of itch. The effect was observed only for the two highest doses of Votucalis (20 and 40 mg kg^−1^), while lower doses of Votucalis (1, 3 and 10 mg kg^−1^) did not show any significant differences between treated and saline control groups (F_(5,41)_ = 3.5, *p* = 0.01). Votucalis did not significantly reduce scratching behaviour caused by chloroquine, showing its ineffectiveness for histamine-independent itch (Figures 1B,D; *p* > 0.05).

### Peripheral Administration of Votucalis Reduced Histaminergic Itch Behavior

**FIGURE 2:**
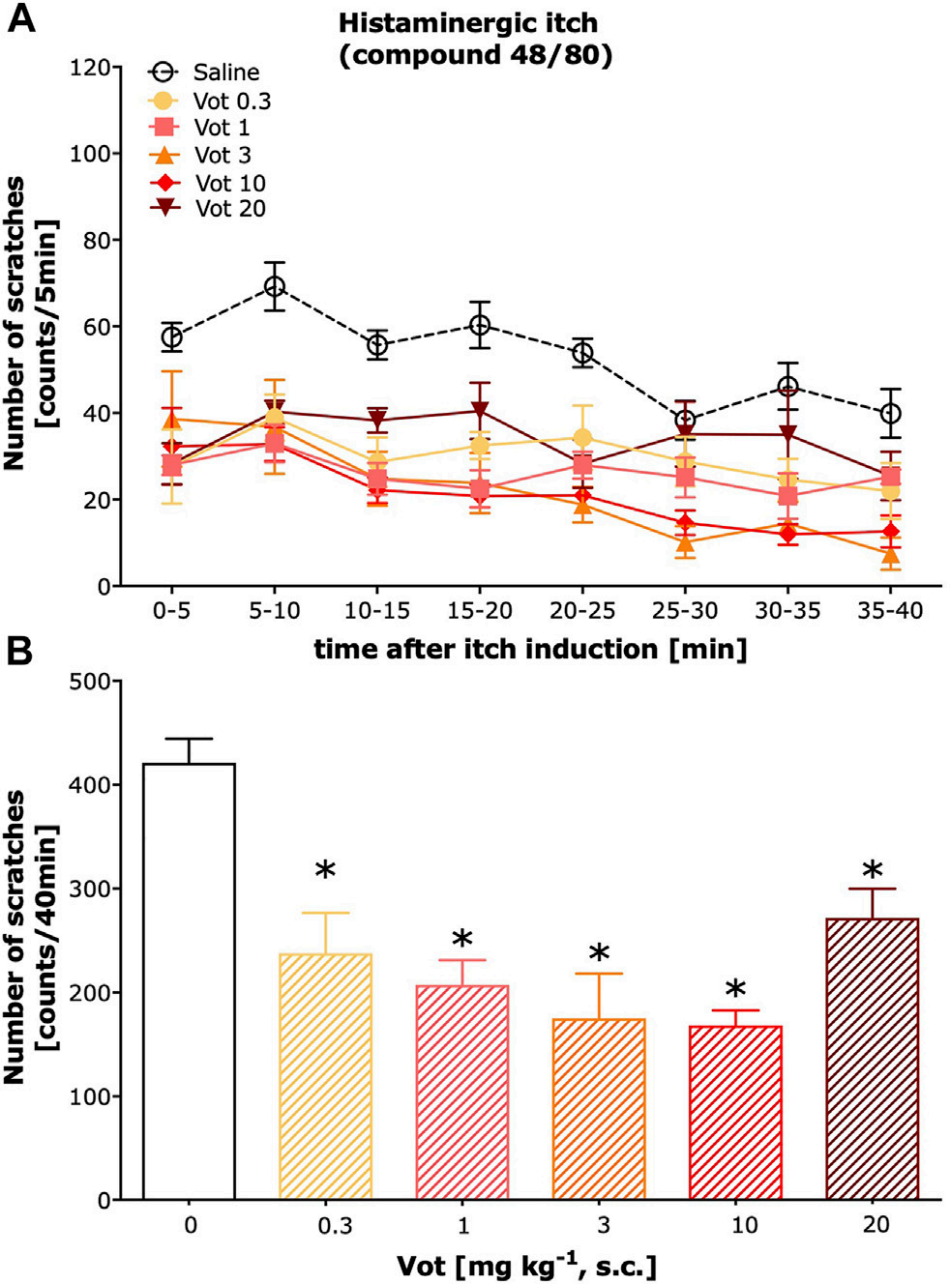
Peripheral administration of Votucalis inhibited itch behaviour induced by histamine-dependent pruritogen in mice. **(A)** Time-course effect of peripheral (s.c.) injection of Votucalis (Vot), or vehicle (saline), on the total number of scratches that occurred over a 40-min time period after injection of compound 48/80 into the nape of the mouse neck. Mice received an injection of Votucalis (0.3–20 mg kg^−1^; *n* = 6) or vehicle (saline; *n* = 17) 30 min before the injection of compound 48/80. Itch behaviour was recorded, and scratches were counted in 5-min intervals for 40 min. **(B)** Bar graph displaying a total number of scratches across a 40-min observation period for each treatment. Data are presented as means ± SEM; the asterisk (*) denotes significance vs*.* vehicle control animals; *p < 0.05 (one-way ANOVA, followed by Bonferroni’s comparison post-hoc test).

Peripheral (s.c.) administration of Votucalis inhibited histamine-dependent itch behaviour induced by compound 48/80 (Figure 2A; drug effect: F_(5,328)_ = 50.43, *p* < 0.0001). This effect was observed over a 40-min observation period in mice indicating a longer duration of action in comparison with the systemic (i.p.) effect produced by Votucalis (Figure 2B; F_(5,41)_ = 14.6, *p* < 0.0001). In addition, the anti-itch effect produced by Votucalis was dose-dependent for the dose range between 0.3 and 10 mg/kg (Figure 2B). However, the highest tested dose of 20 mg kg^−1^ showed decreased anti-itch effect compared to the lower doses of Votucalis tested, although the effect was still significant when compared with the saline control (Figure 2B; *p* = 0.003). This observation indicates a biphasic or bell-shape dose-response curve produced by peripherally administered Votucalis.

### Peripheral Histamine H_1_ and H_2_ Receptors as Well as Central Histamine H_4_ Receptors Potentiated the Effects of Votucalis

**FIGURE 3:**
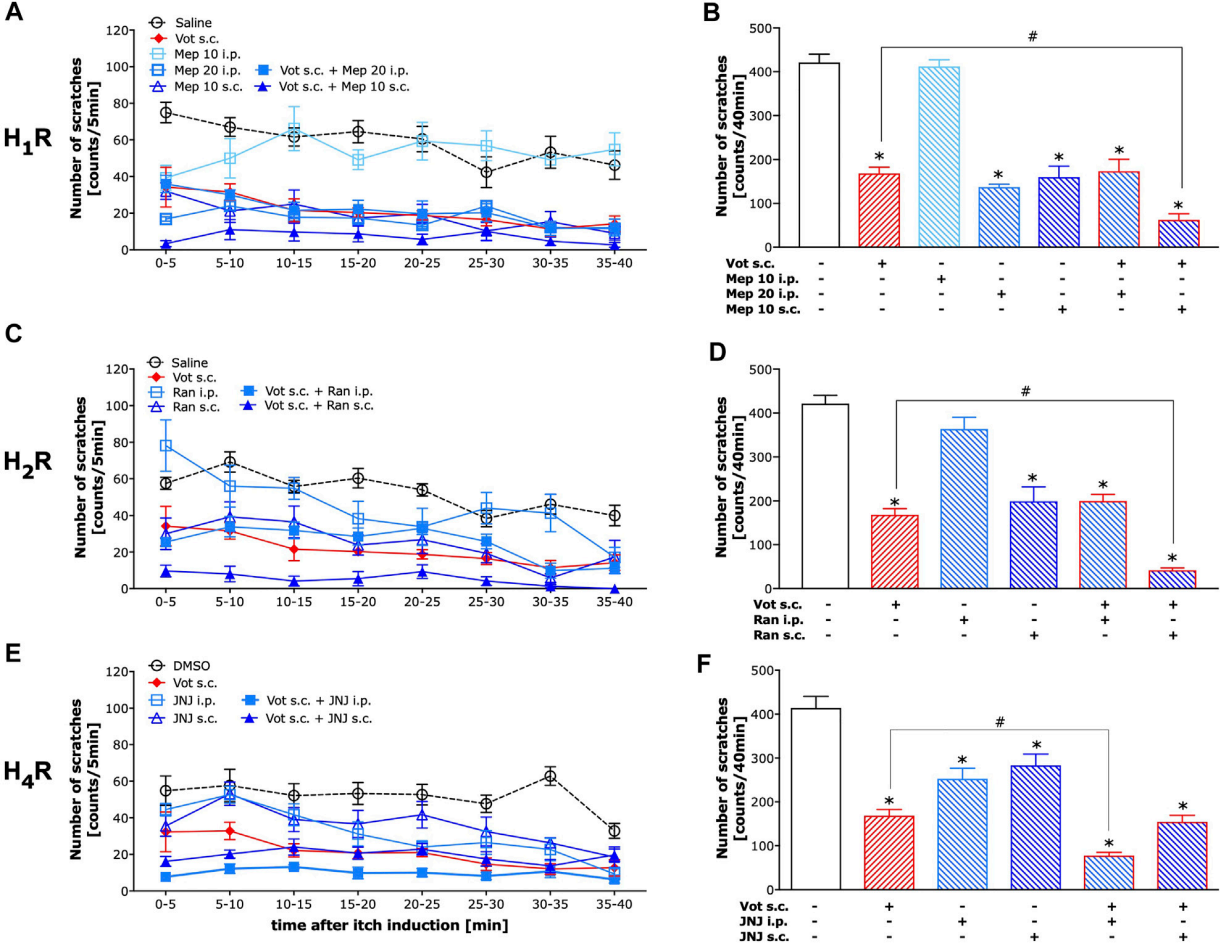
Peripheral H_1_ and H_2_ receptors, as well as central H_4_ receptors, attenuated the anti-itch effect induced by Votucalis in mice. Left panel **(A,C,E)** Time-course effect of injection of Votucalis (Vot, 10 mg kg^−1^; s.c.; *n* = 9; A, C, E), H_1_ receptor (H_1_R) antagonist mepyramine (Mep, 10–20 mg kg^−1^; i.p. or s.c.; *n* = 6; A), H_2_ receptor (H_2_R) antagonist ranitidine (Ran, 15 mg kg^−1^; i.p. or s.c.; *n* = 6; B), H_4_ receptor (H_4_R) antagonist JNJ 7777120 (JNJ, 20 mg kg^−1^; i.p. or s.c.; *n* = 6; C) or vehicle (saline or DMSO, *n* = 6–17, **A,C,E**), on the total number of scratches that occurred over a 40-min time period after injection of compound 48/80 into the nape of the mouse neck. Mice received a single injection of each of the drugs or a combination of Votucalis with one of the histamine receptor antagonists or vehicle 30 min before the injection of pruritic agent. Itch behaviour was recorded, and scratches were counted in 5-min intervals for 40 min. Right panel **(B,D,F)** Bar graphs displaying a total number of scratches across the 40-min observation period for each treatment. Data are presented as means ± SEM, *n* = 6–17 in each group. The drugs administered to each group is indicated below the respective bar. The asterisk (*) denotes significance *vs.* vehicle control animals; *p < 0.05 (one-way ANOVA, followed by Bonferroni’s comparison post-hoc test). The hash (#) denotes significance vs*.* Votucalis treated animals; #*p* < 0.05 (one-way ANOVA, followed by Bonferroni’s comparison post-hoc test).

As illustrated in Figures 3A,B, mepyramine, a selective H_1_ receptor antagonist, when injected systemically (i.p.) required a high dose to reduce histamine-dependent itch produced by compound 48/80 as 10 mg kg^−1^ was ineffective (*p* > 0.05), while 20 mg kg^−1^ significantly inhibited compound 48/80-induced itch (*p* < 0.0001). In contrast, peripheral (s.c.) administration of mepyramine at the dose of 10 mg kg^−1^ was significantly effective in inhibiting of compound 48/80-induced itch (*p* < 0.0001). This peripherally effective dose of mepyramine when co-administered with Votucalis (10 mg kg^−1^, s.c.) produced stronger inhibition of histamine-dependent itch induced by compound 48/80 (*p* = 0.007) when compared to the effect produced by the drugs alone. Systemic administration of mepyramine (20 mg kg^−1^, i.p.) together with Votucalis (10 mg kg^−1^, s.c.) did not attenuate the anti-itch effect of the drugs alone (*p* > 0.05).

As illustrated in Figures 3C,D, ranitidine, a selective H_2_ receptor antagonist, when injected systemically (i.p.) at the dose of 15 mg kg^−1^ did not reduce histamine-dependent itch produced by compound 48/80 (*p* > 0.05). The same dose of ranitidine when injected peripherally (s.c.) significantly reduced compound 48/80-induced itch (*p* < 0.0001), as well as the anti-itch effect produced by peripherally co-administered Votucalis (10 mg kg^−1^, s.c.) and ranitidine (15 mg kg^1-^, s.c.) was stronger when compared to the effect produced by the drugs alone (*p* = 0.002). Systemic administration of ranitidine (15 mg kg^1-^, i.p.) together with Votucalis (10 mg kg^−1^, s.c.) did not attenuated the anti-itch effect when compared to the effect produced by the drugs alone (*p* > 0.05).

As illustrated in Figures 3E,F, JNJ 7777120, a selective H_4_ receptor antagonist, when injected either systemically (i.p.) or peripherally (s.c.) at the dose of 20 mg kg^−1^ significantly reduced histamine-dependent itch produced by compound 48/80 (*p* < 0.0001 and *p* = 0.0004 respectively). However, only systemically administered JNJ 7777120 (20 mg kg^−1^, i.p.) when co-administered with Votucalis (10 mg kg^−1^, s.c.) produced stronger inhibition of compound 48/80-induced itch (*p* = 0.002), while peripheral administration of JNJ 7777120 (20 mg kg^−1^, s.c.) did not attenuate the anti-itch effect when co-administered with Votucalis (10 mg kg^−1^, s.c.; *p* > 0.05).

In summary, Votucalis provided inhibition of histaminergic itch around 62%, which was further increased by both peripheral H_1_ and H_2_ or central H_4_ antagonism up to around 85, 90 and 81%, respectively. Peripheral H_1_ and H_2_ or central H_4_ antagonism without Votucails inhibited histaminergic itch around 64, 53 and 27%, respectively. The incomplete inhibition of itch by Votucalis is assumed to be due to high levels of histamine released by mast cell degranulation in this model.

### Systemic and Peripheral Administration of Votucalis Reduced Mechanical Hypersensitivity

**FIGURE 4:**
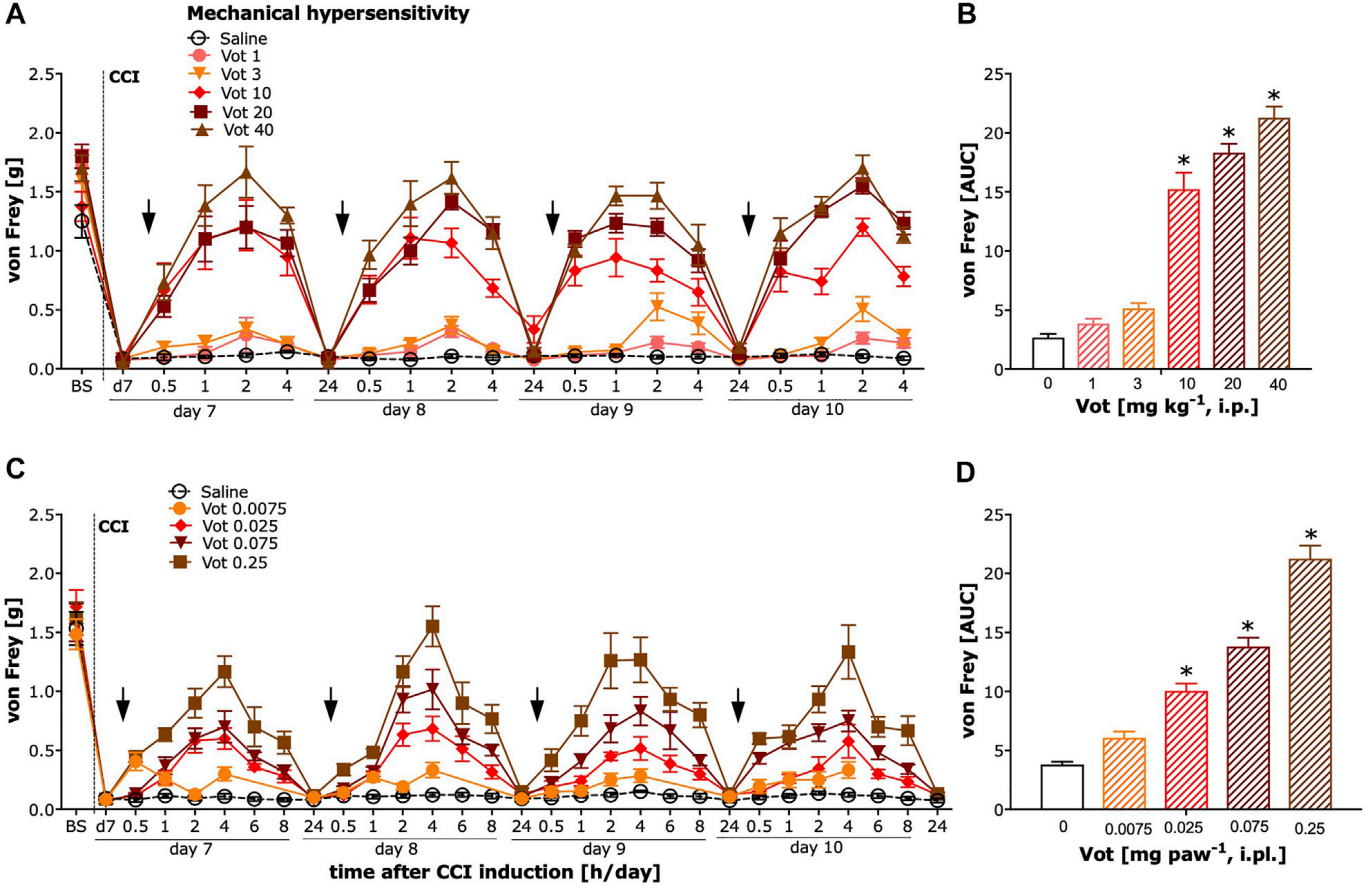
Chronic systemic and peripheral administration of Votucalis significantly attenuated mechanical hypersensitivity in the chronic constriction injury (CCI) model of neuropathic pain in mice. Left panel **(A,C)** Time-course effect of systemic (i.p.) injection of Votucalis (Vot, 1–40 mg kg^−1^, *n* = 6; A) and peripheral (i.pl.) injection of Votucalis (Vot, 0.0075–0.25 mg paw^−1^, *n* = 6–12; C) administered into the plantar surface of the ipsilateral paw to injury, or vehicle (saline, *n* = 6–14; A, C), on the mechanical withdrawal threshold measured with von Frey filaments. The measurements were assessed before injury as basal pain threshold (BS), and then 7 days following the injury (d7). The effect of Votucalis was assessed 0.5–24 h after each of four consecutive once daily (arrow) intraperitoneal **(A)** or intraplantar **(C)** injections. Data are presented as means ± SEM, *n* = 6–14 in each group. Right panel **(B,D) (B)** The area under the curve (AUC) summarizing measurements in A. **(D)** The area under the curve (AUC) summarizing measurements in C. The asterisk (*) denotes significance vs*.* vehicle control animals; *p < 0.05 (one-way ANOVA, followed by Bonferroni’s comparison post-hoc test).

The analgesic effectiveness of systemic (i.p.) administration of Votucalis was assessed by measuring the paw withdrawal threshold in response to mechanical stimuli using von Frey filaments in mice with neuropathic pain (CCI model). After systemic administration of Votucalis, the mechanical hypersensitivity in CCI mice was significantly reduced (Figure 4A; drug effect: F (5,812) = 300.8, *p* < 0.0001) in a dose dependent manner. The three highest doses of Votucalis, 10, 20 and 40 mg kg^−1^, significantly attenuated mechanical hypersensitivity compared to the saline control (Figure 4B; F_(5,32)_ = 102.2, *p* < 0.0001). The greatest reduction in mechanical hypersensitivity was observed 1 and 2 h after the first injection of systemic Votucalis (10, 20 and 40 mg kg^−1^), when compared with saline controls, and this effect was maintained at 4 h after the administration, but 24 h after the administration, was no longer observed (Figure 4A). A similar pattern of analgesic effect was observed after each of four consecutives daily systemic administration of Votucalis (10, 20 and 40 mg kg^−1^; Figure 4A), indicating lack of pharmacological tolerance. No significant difference in mechanical hypersensitivity was seen with the lowest doses of Votucalis, 1 and 3 mg kg^−1^, when compared to the saline control (Figures 4A,B; *p* > 0.05).

The analgesic efficacy of peripheral (i.pl.) administration of Votucalis was assessed by measuring the paw withdrawal threshold in response to mechanical stimulus using von Frey filaments in CCI mice. After peripheral administration of Votucalis, the mechanical hypersensitivity in CCI mice was significantly reduced with the three highest doses 0.025, 0.075 and 0.25 mg paw^−1^ when compared to vehicle control (Figures 4C,D; F_(4,25)_ = 93.38, *p* < 0.0001). The greatest reduction in mechanical hypersensitivity was observed 4 h after the first injection of Votucalis (0.025, 0.075 and 0.25 mg paw^−1^), when compared with saline controls and this effect gradually declined, and 24 h after the administration was no longer observed (Figure 4C). A similar pattern of analgesic effect was observed after each of the four consecutive daily i.pl. administrations of Votucalis (0.025, 0.075 and 0.25 mg paw^−1^; Figure 4C), indicating a lack of pharmacological tolerance. No significant difference in mechanical hypersensitivity was seen with the lowest dose of Votucalis, 0.0075 mg paw^−1^, when compared to the saline control (Figures 4C,D; *p* > 0.05).

Sham mice, after systemic (i.p.) or peripheral (i.pl.) administration of Votucalis (40 mg kg^−1^ or 0.25 mg paw^−1^) showed a lack of change in paw withdrawal threshold (Figures 6A,B; *p* > 0.05). Only a weak significant effect was observed after localised peripheral administration of Votucalis into the ipsilateral paw (to CCI), when the analgesic effect was assessed on the contralateral paw (Figure 6C; t_(11)_ = 3.4, *p* = 0.006).

### Systemic, but Not Peripheral, Administration of Votucalis Produced a Weak Effect on Neuropathic Pain-Induced Thermal (Heat) Hypersensitivity

**FIGURE 5:**
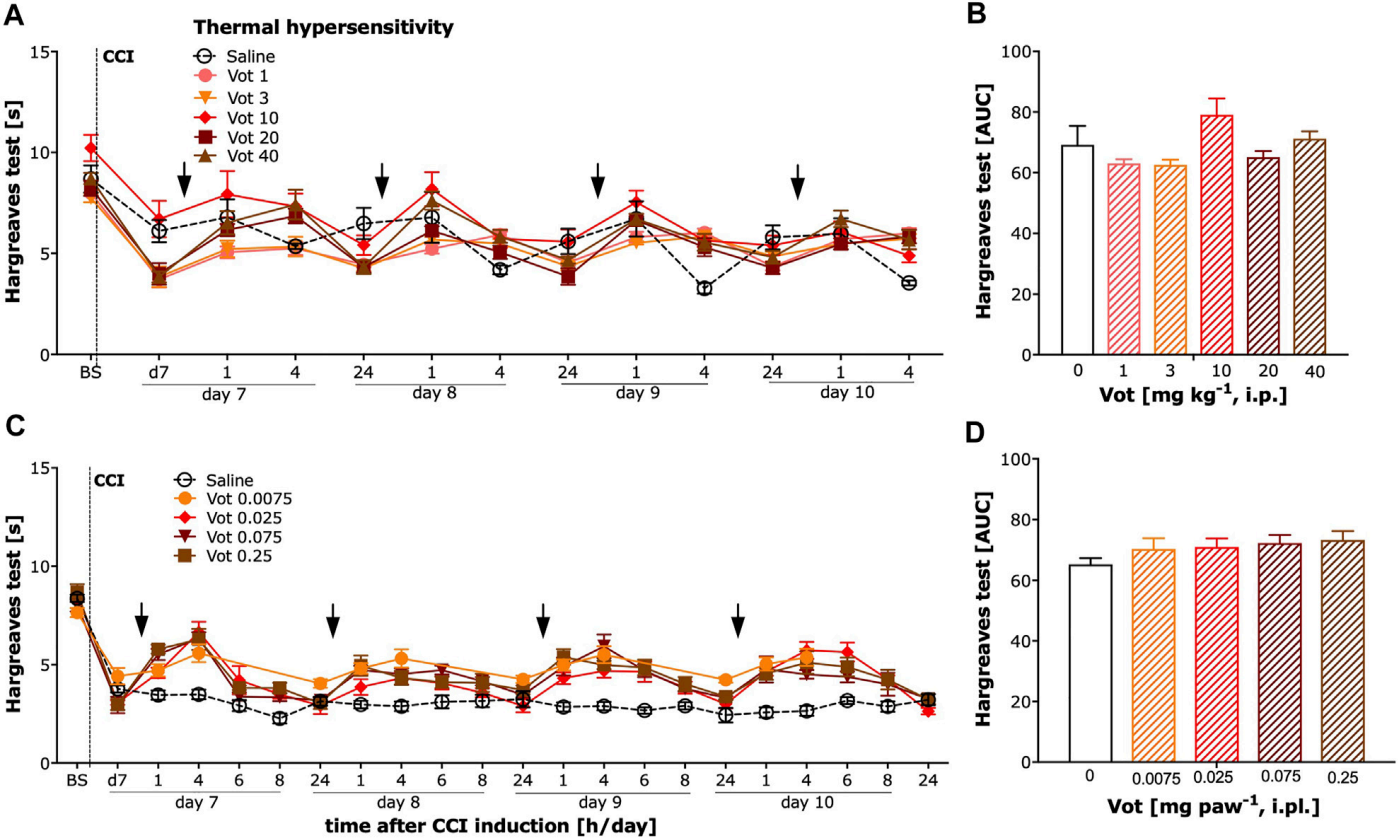
Chronic systemic administration of Votucalis produced a weak effect while peripheral administration of Votucalis did not attenuate thermal (heat) hypersensitivity in the chronic constriction injury (CCI) model of neuropathic pain in mice. Left panel **(A,C)** Time-course effect of systemic (i.p.) injection of Votucalis [Vot, 1–40 mg kg^−1^, *n* = 6; **(A)]** and peripheral (i.pl.) injection of Votucalis [Vot, 0.0075–0.25 mg paw^−1^, *n* = 6; **(C)]** administered into the plantar surface of the injured paw, or vehicle [saline, *n* = 6; **(A,C)]**, on the thermal (heat) withdrawal threshold measured with the Hargreaves test. The measurements were assessed before injury as basal pain threshold (BS), and then 7 days following the injury (d7). The effect of Votucalis was assessed 0.5–24 h after each of four consecutive once daily (arrow) intraperitoneal **(A)** or intraplantar **(C)** injections. Data are presented as means ± SEM. *Right panel* **(B,D) (B)** The area under the curve (AUC) summarizing measurements in A. **(D)** The area under the curve (AUC) summarizing measurements in C. One-way ANOVA in B *p > 0.05 but Bonferroni’s comparison post-hoc test non-significant vs. vehicle control animals. In D lack of significance vs. vehicle control animals.

The analgesic efficacy of systemic (i.p.) and peripheral (i.pl.) administration of Votucalis was also assessed by measuring the paw withdrawal latency in response to heat stimuli using the Hargreaves test in CCI mice. Systemic administration of Votucalis produced a weak effect on heat hypersensitivity (Figures 5A,B; F_(5,32)_ = 2.8, *p* = 0.03, Bonferroni’s comparison post-hoc test non-significant). However, no significant difference in heat hypersensitivity was observed at any doses of Votucalis tested after peripheral administration (Figures 5C,D; F _(4,25)_ = 1.2, *p* = 0.34).

**FIGURE 6:**
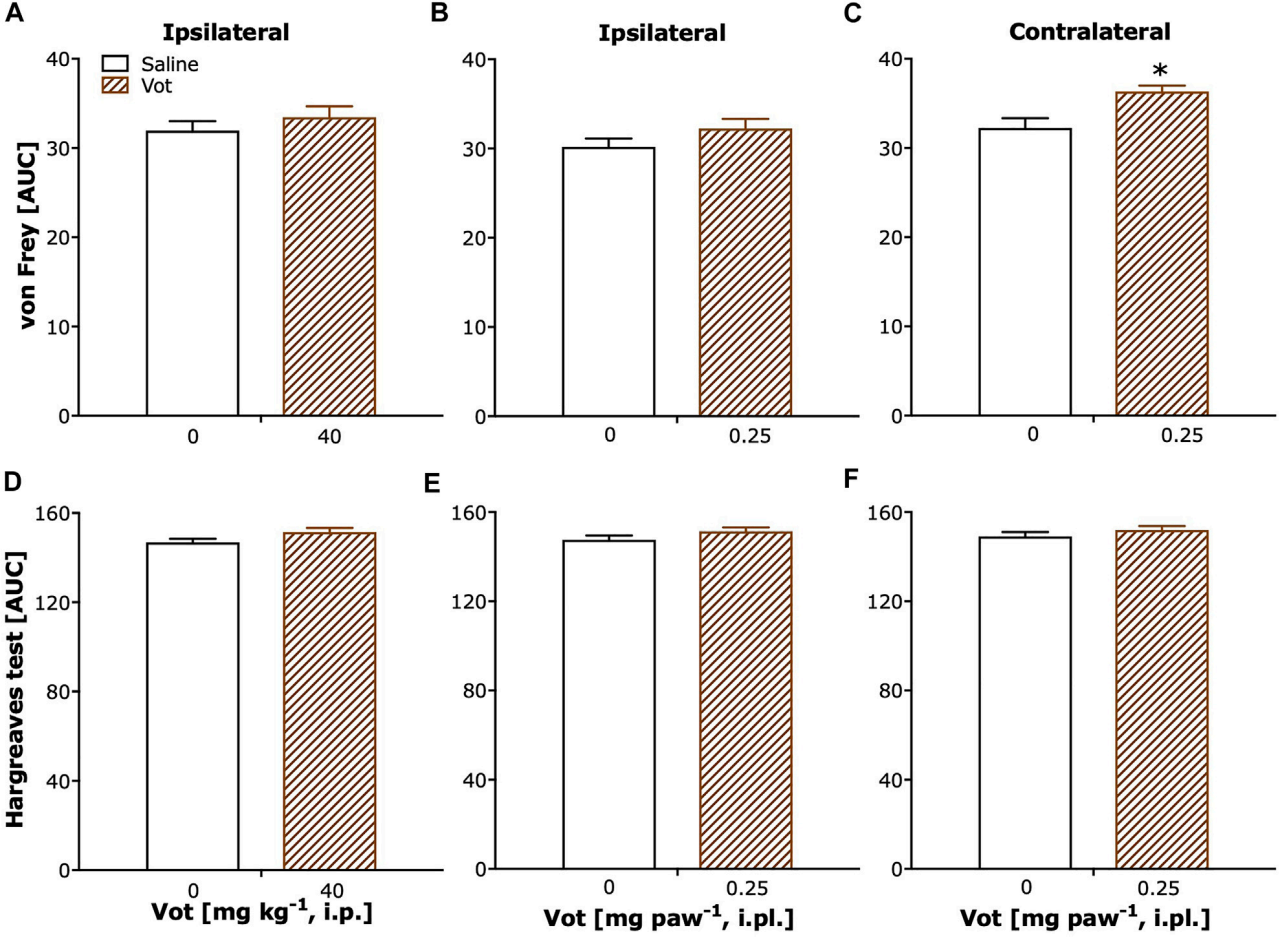
Chronic systemic and peripheral administration of Votucalis did not attenuate overall mechanical or thermal sensitivity in sham mice. **(A–C)** The area under the curve (AUC) summarizing time-course effect of systemic (i.p.) injection of Votucalis [Vot, 40 mg kg^−1^, *n* = 8; **(A)]** and peripheral (i.pl.) injection of Votucalis [Vot, 0.25 mg paw^−1^, *n* = 8; **(B,C)**], or vehicle [saline, *n* = 5; **A–C)**], on the mechanical withdrawal threshold measured with von Frey filaments. **(D–F)** The area under the curve (AUC) summarizing time-course effect of systemic (i.p.) injection of Votucalis [Vot, 40 mg kg^−1^, *n* = 8; **(D)**] and peripheral (i.pl.) injection of Votucalis [Vot, 0.25 mg paw^−1^, *n* = 8; **(E,F)**], or vehicle [saline, *n* = 5; **(D–F)**, on the thermal withdrawal threshold measured with the Hargreaves test. The measurements were taken on the ipsilateral paw **(A,B,D,E)** or contralateral paw **(C, F)** similarly as described in Figures 4, 5 (detailed data not shown). The asterisk (*) denotes significance *vs.* vehicle control animals; *p < 0.05 (two-tailed unpaired *t*-test).

No change in paw withdrawal latency to heat stimuli was observed in sham mice after either systemic or peripheral (i.pl.) administration of Votucalis (40 mg kg^−1^ or 0.25 mg paw^−1^) in ipsilateral or contralateral paws (Figures 6D–F; *p* > 0.05).

Votucalis, in any of the tested doses administered, either systemically or peripherally, did not cause any form of visible discomfort throughout the period of 4 days, and there were no significant changes in mouse body weights during this study (data not shown).

### Peripheral Administration of Votucalis Produced Anti-pruritic and Anti-nociceptive Effects at a Lower Dose Range Compared to Systemic Administration

**FIGURE 7:**
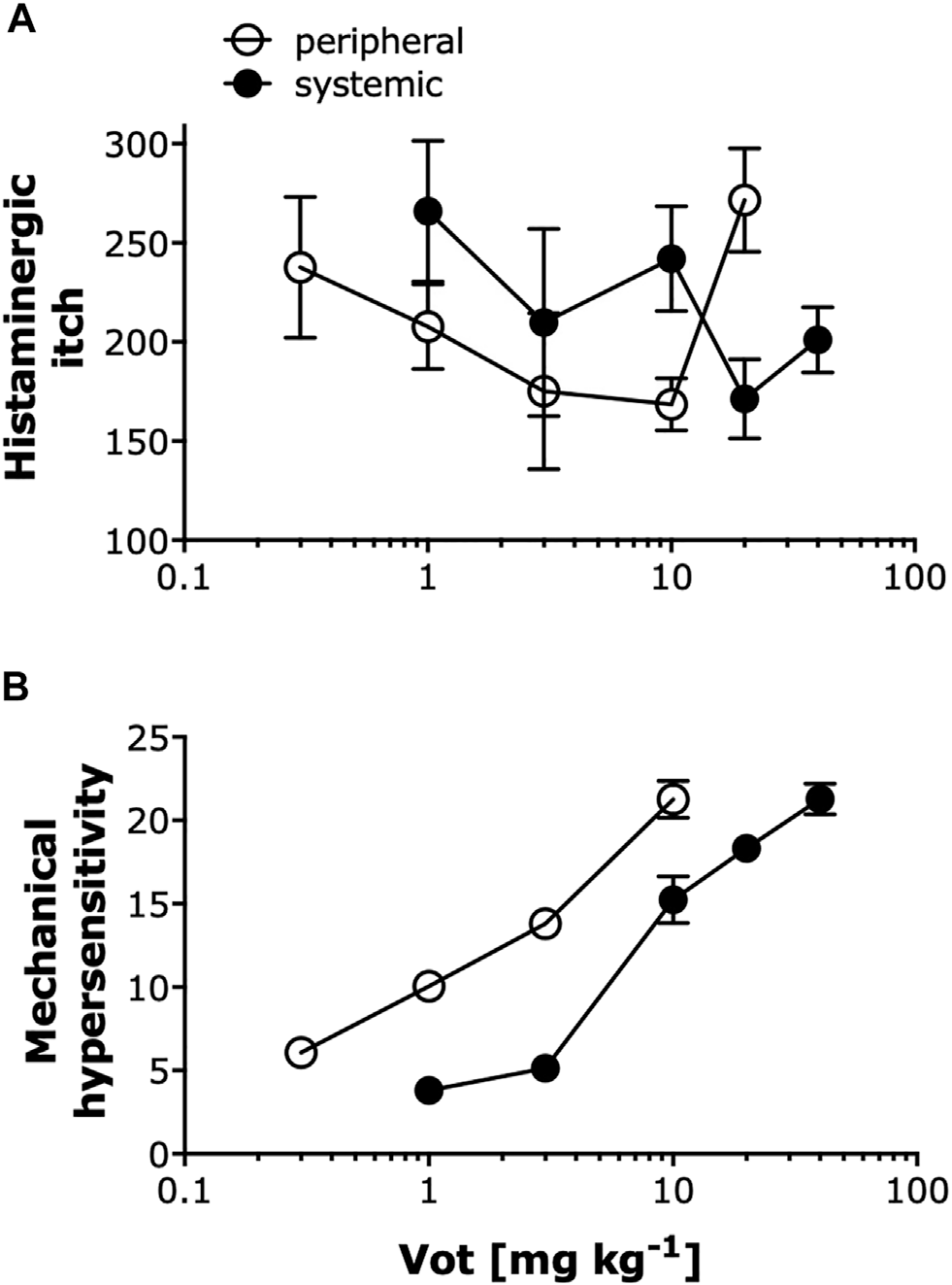
Peripheral administration of Votucalis produced anti-pruritic and anti-nociceptive effects at a lower dose range compared to systemic administration. **(A)** Dose-response effect of Votucalis (Vot, 0.3–40 mg kg^−1^; i.p. or s.c.) in histamine-dependent itch induced by injection of compound 48/80. Points represent the total number of scratches across a 40-min observation period for each treatment based on the data presented in Figures 1, 2. Data are presented as means ± SEM, *n* = 6–18 in each group. **(B)** Dose-response effect of Votucalis (Vot, 0.3–40 mg kg^−1^; i.p. or i.pl.) in the chronic constriction injury (CCI) model of neuropathic pain. Points represent the area under the curve (AUC) presented in Figure 4 and referring to changes in the mechanical withdrawal threshold measured with von Frey filaments. Data are presented as means ± SEM, *n* = 6–14 in each group.

As summarized in Figure 7, Votucalis produced dose-dependent anti-itch effects on histamine-dependent itch produced by compound 48/80 (Figure 7A), and anti-nociceptive effects on mechanical hypersensitivity resulting from injury of the sciatic nerve and subsequent development of neuropathic pain (Figure 7B). Overall, peripheral (s.c. or i.pl.) administration of Votucalis produced anti-itch and anti-nociceptive effects at a lower dose range in comparison to systemic (i.p.) administration indicating higher potency after peripheral transdermal Votucalis administrations observed as a left-ward shift of the dose-response curve for Votucalis.

### Peripheral Administration of Votucalis did Not Affect Short-Term Recognition Memory

**FIGURE 8:**
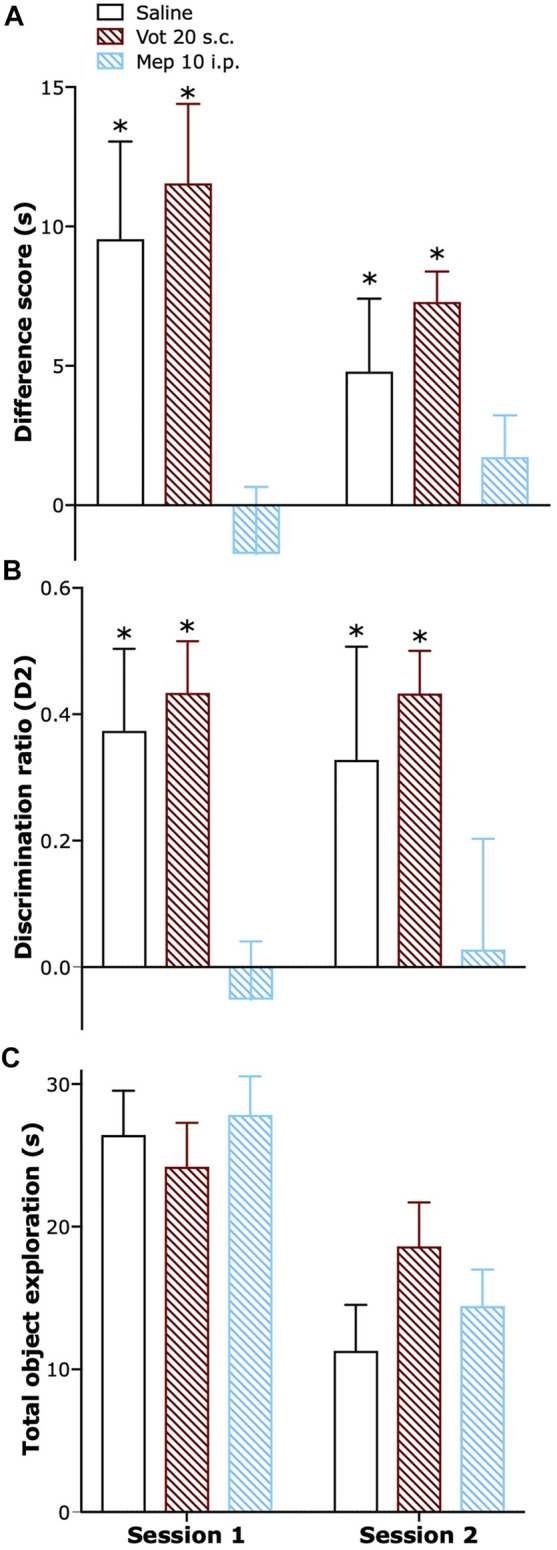
Peripheral administration of Votucalis did not affect short-term recognition memory in naïve mice. **(A)** Time-course effect of a single (session 1) and chronic (session 2) peripheral (s.c.) injection of Votucalis (Vot), or systemic (i.p.) injection of H_1_ receptor (H_1_R) antagonist mepyramine (Mep) or vehicle (saline), on the performance in the spontaneous novel object recognition task. Mice received a single or chronic (across four consecutive days) injection of Votucalis (20 mg kg^−1^; *n* = 8), mepyramine (10 mg kg^−1^; *n* = 8) or vehicle (saline; *n* = 8) 30 min before the task; behavior was recorded and scored offline. Performance in the task is shown as a difference score (mean time spent exploring the novel objects minus the mean time spent exploring the familiar objects) in session 1 and 2. Scores above zero indicate a novelty preference. **(B)** Performance in the task is also shown as a discrimination ratio (difference score divided by mean total time spent exploring the objects in the test phase) for session 1 and 2. **(C)** Total duration of object exploration in the test phase for session 1 and 2. Data are presented as means ± SEM, *n* = 8 in each group. The asterisk (*) denotes significance vs. mepyramine treated animals; *p < 0.05 (one-way ANOVA, followed by Bonferroni’s comparison post-hoc test).

Single peripheral (s.c.) administration of Votucalis preserved short-term recognition memory in naïve mice while systemic (i.p.) administration of H_1_ receptor antagonist mepyramine resulted in a significant impairment in short-term recognition memory. Similar effect was also observed when Votucalis and mepyramine were administered across four consecutive days (Figures 8A,B; drug effect: F_(2,21)_ = 12.2 *p* = 0.0003 and F_(2,21)_ = 10.05 *p* = 0.0009, respectively). Administration of both Votucalis and mepyramine had no visible effect on the total time the animals spent exploring the objects (Figure 8C; *p* > 0.05).

In addition, systematic (i.p.) administration of Votucalis (20 mg kg^−1^), either single or chronic, did not impair short-term recognition memory and had no effect on the balance and coordination of naïve mice as measured using the rotarod test (*p* > 0.05; data not shown).

## Discussion

In this study, we demonstrated that Votucalis, a novel CNS-sparing and high-affinity recombinant histamine binding protein, produced both anti-pruritic and anti-nociceptive effects in mouse models of acute itch and chronic neuropathic pain following both systemic and peripheral administration, which represent commonly used routes of drug applications in clinical practice. The main finding, however, highlights the advantage of peripheral transdermal administration of Votucalis, as the anti-pruritic and anti-nociceptive effects were achieved at significantly lower doses and produced longer duration of effect than systemic administration. Peripheral route of administration was also free from CNS-mediated (side) effects. Thus, this observation provides the first evidence for therapeutic targeting peripherally released histamine by Votucalis to deliver a novel strategy (histamine capture) for potentially safer and more efficacious control of conditions that are known to be regulated by histamine.

### Votucalis Attenuated Histaminergic Itch

Our study showed that both systemic and peripheral transdermal administrations of Votucalis attenuated scratching behaviour associated with activation of a histamine-dependent mechanism, as compound 48/80 is a well-known inducer of histamine-dependent itch (Inagaki et al., 2002; Obara et al., 2015). Intracutaneous injection of this compound is known to cause mast cells degranulation resulting in histamine release from human, dog and rodent cutaneous mast cell in a concentration-dependent fashion *via* a mechanism involving activation of histamine receptors. Activation of all these receptors expressed in primary sensory neurons results in the activation of phospholipase A2 (PLA2), phospholipase C-β3 (PLCβ3) and protein kinase C δ (PCKδ) leading to increase of intracellular Ca^2+^ in primary sensory neurons and DRG neurons *via* transient receptor potential subfamily (TRPA1/V1) (Barrett et al., 1985; Tomoe et al., 1992; Bell et al., 2004; Kim et al., 2004; Shim et al., 2007; Liu et al., 2013; Wilzopolski et al., 2021). Histamine released from activated mast cells locally within the dermis, causes leakage of plasma into surrounding tissues and cellular infiltration of neutrophils and eosinophils (Tiligada and Ennis, 2020). Also, in experimental conditions intradermal injection of histamine was shown to produce itch in humans and rodents (Heyer et al., 1997; Laidlaw et al., 2002). Since the crystal structure of Votucalis revealed a high-affinity site for histamine binding (Paesen et al., 1999; Paesen et al., 2000), it is very likely that Votucalis neutralised endogenously released histamine, resulting in a potent and dose-dependent anti-itch effects. The anti-itch effect of Votucalis was particularly profound after peripheral transdermal administration. Specifically, subcutaneous delivery of Votucalis directly to the area affected by itch was effective over the whole 40-min observation period, at a dose over 60-fold lower compared to the lowest systemic dose, that was effective; notably this was only effective during the first 20 min from induction of itch. The striking difference in the potency and efficacy of Votucalis observed when the drug was administered transdermally to the area of itch sensation vs. systemically, further emphasises the nature of Votucalis, as a non-brain-penetrating, highly potent histamine local scavenger. It seems likely that the ability of Votucalis to sequester histamine, subsequently suppressed the binding of histamine to all histamine receptors potentially leading to decrease in firing and excitability of the itch-specific primary afferents, in particular Aδ- and C-fibres, that resulted in attenuation of histaminergic itch (LaMotte et al., 2014; Obara et al., 2015). To further confirm that the anti-itch effect of Votucalis was mediated by neutralisation of histamine, we identified that Votucalis did not reduce scratching behaviour caused by chloroquine that is a Mas-related G protein-coupled receptor A3 (MrgprA3) agonist that upon intracutaneous administration elicits scratching behavior resulting from activation of MrgprA3+ primary sensory neurons that were shown to be essential for itch sensation (Shiratori-Hayashi et al., 2019). Thus, we showed Votucalis ineffectiveness for histamine-independent itch. However, it should be noted that the highest dose of Votucalis used to inhibit itch was less effective compared to the efficacy of lower doses of Votucalis, potentially revealing a biphasic or bell-shape dose response curve produced by Votucalis. Given that histamine plays very diverse physiological roles, this observation may suggest that higher doses of Votucalis may activate mechanisms that conversely promote itch sensation (possibly *via* the H_3_ receptor) and, therefore, counteract the scavenging ability of Votucalis. This may require further investigation. Note, that the anti-itch effects of Votucalis was incomplete, consistent with the high non-physiological levels of histamine released by compound 48/80 in the itch study.

### Peripheral H_1_ Receptor, Peripheral H_2_ Receptor and Central H_4_ Receptor Mediated Histaminergic Itch as Well as Votucalis Anti-Itch Effects

H_1_ receptor antagonists are widely used to relieve itch, however their therapeutic efficacy is limited (Shim and Oh, 2008), indicating that different histamine receptors may be involved in the mediation of itch (Kollmeier et al., 2014). Herein, by using selective histamine receptors antagonists and different routes of their administration we were able to distinguish, for the first time, the potential involvement of peripheral and central histamine receptors in itch. Our study indicates that histaminergic itch is predominantly mediated by peripheral H_1_ and H_2_ receptors, and less by central H_1_ and H_2_ receptors, as peripherally administered selective antagonists of these receptors inhibited histaminergic itch at doses that were not effective with systemic administration. Thus, this may suggest the involvement of predominantly peripheral H_1_ and H_2_ receptors however further investigations may be required to confirm this effect. Nevertheless, these findings provide new insight into the mechanism of the H_1_ receptor mediated anti-itch effect as some studies have argued that the anti-itch effect resulting from H_1_ antagonism is due to sedation, rather than the direct blockade of H_1_ receptor on sensory neurons (Imaizumi et al., 2003; Bell et al., 2004). In line with our observation, both mRNA and functional expression of H_1_ receptor were shown on peripheral neurons displaying characteristics of C-fibers (Kashiba and Senba, 2001; Rossbach et al., 2011). In addition, Bell et al. (2004) found that intradermal administration of a H_1_ agonist caused dose-dependent scratching in mice.

Our findings also extend the understanding of the role of H_2_ receptors in itch, as the available literature is rather inconclusive (Bell et al., 2004; Rossbach et al., 2011). It seems that H_2_ receptor-mediated anti-itch response is due to antagonism of predominantly peripherally expressed H_2_ receptors that are known to be present on primary afferent neurons (Kajihara et al., 2010), although their functional expression has not yet been confirmed (Rossbach et al., 2011). The proposed role of peripheral H_1_ and H_2_ receptors in itch transmission was further emphasised by the use of Votucalis as the anti-itch effects was stronger when antagonists targeting peripheral H_1_ and H_2_ receptors were co-administered with the drug.

In line with other *in vivo* studies, including experiments using knockout animals, we found that antagonism at H_4_ receptor suppressed histaminergic itch (Dunford et al., 2007; Rossbach et al., 2011; Wilzopolski et al., 2021). Interestingly, this effect was observed after both peripheral and systemic administration of a H_4_ receptor antagonist, however only systemically administered H_4_ antagonist produced stronger anti-itch effect when co-administered with Votucalis. We, therefore, suggest that central H_4_ receptors may be predominantly involved in the regulation of histaminergic itch.

### Votucalis Attenuated Mechanical Neuropathic Pain

Our study showed that both systemic and peripheral transdermal administrations of Votucalis almost completely blocked mechanical hypersensitivity in neuropathic mice. Similarly, as in the itch study, the anti-nociceptive effect of Votucalis was most potent after peripheral transdermal administration; the lowest anti-nociceptive peripheral effective dose was 10-fold lower in comparison to the lowest effective systemic dose. This Votucalis-induced effect highlights the importance of histamine for the maintenance of neuropathic pain symptoms in the periphery where histamine is known to produce nociceptive effects (Yue et al., 2014; Khalilzadeh et al., 2018). Indeed, as a result of tissue injury or damage, histamine released from neuronal and non-neuronal cells, in close proximity to sensory fibers, contributes to the development and maintenance of mechanical and thermal hypersensitivity *via* sensitizing peripheral polymodal nociceptors, which results in increased firing rates and generate action potentials in the neurons (Khalilzadeh et al., 2018; Obara et al., 2020). In addition, it was shown that histamine contributes to neuropathic pain mechanism by increasing voltage-gated Na^+^ channels, in particular Nav1.8 and Nav1.9 expression in primary afferent neurons and L4/L5 DRG neurons (Yue et al., 2014; Bennett et al., 2019). Thus, it seems that Votucalis efficiently neutralized endogenously released histamine causing a potent and dose-dependent anti-nociceptive effect. Interestingly, however, while mechanical hypersensitivity was blocked by peripherally administered Votucalis, heat hypersensitivity remained unaffected by this treatment. This modality-specific anti-nociceptive effect may suggest involvement of H_3_ receptors and that Votucalis prevents histamine binding to H_3_ receptor since both pharmacological and genetic manipulations of H_3_ receptor activity have confirmed its importance and specificity for mechanical hypersensitivity (Cannon et al., 2003; Wei et al., 2016). In addition, anatomical studies have confirmed localization of H_3_ receptors on Aδ-fibers that conduct tactile sensation (Lawson, 2002; Cannon et al., 2007) as well as the ability of H_3_ antagonists to block secondary mechanical hypersensitivity (Medhurst et al., 2007). It may suggest that Votucalis, by scavenging endogenous histamine, reduced the sensitivity of H_3_-positive A-fibers resulting in a diminished input to the dorsal horn, supporting the potential role for H_3_ receptors in the modulation of central sensitization. In contrast, heat hypersensitivity is regarded as a sign of the peripheral sensitization of C-fibers, which do not express H_3_ receptors (Cannon et al., 2007). This C-fibre sensitization may be due to the involvement of H_4_ receptors (Obara et al., 2020).

In summary, this is the first report showing that targeting histamine itself, by sequestering the endogenous ligand within Votucalis, may represent a new tool to control conditions that are known to be regulated by peripherally released histamine. It should be, however, noted that future studies should attempt to quantify the efficacy of histamine scavenging by Votucalis. Nevertheless, *in vivo* neutralization of locally administered Votucalis has also shown previously a wide range of anti-inflammatory effects in mouse models of acute respiratory distress syndrome (ARDS) or allergic asthma (Ryffel et al., 2005). Herein, our new approach may provide many therapeutic advantages over drugs targeting histamine receptors, which when tested for their utility in attenuating itch and pain, have shown inconsistent and limited efficacy. Our studies, using the spontaneous novel object recognition task, suggest that local peripheral sequestration of histamine by Votucalis may have therapeutic potential as a non-sedating and non-addictive analgesic agent. The use of analgesics that act at the peripheral level is justified in accordance with current concepts in pain medicine, which emphasize the importance of an individualized and mechanism-based approach in pain management (Müller-Schwefe et al., 2017; Kocot- Kępska et al., 2021). It is expected that peripherally (e.g., transdermally) applied analgesics will only target the underlying molecular/cellular mechanisms in the periphery, negating the need to consider systemic mechanisms and, therefore, their long-term use may be safer, as well as effective.

## Data Availability

The raw data supporting conclusion of this article is available at https://doi.org/10.25405/data.ncl.19203461.
